# A multi-state model of chemoresistance to characterize phenotypic dynamics in breast cancer

**DOI:** 10.1038/s41598-018-30467-w

**Published:** 2018-08-13

**Authors:** Grant R. Howard, Kaitlyn E. Johnson, Areli Rodriguez Ayala, Thomas E. Yankeelov, Amy Brock

**Affiliations:** 1 Department of Biomedical Engineering, The University of Texas at Austin, Austin, Texas 78712 USA; 2 Department of Chemical Engineering, The University of Texas at Austin, Austin, Texas 78712 USA; 3 Institute for Computational Engineering Sciences, The University of Texas at Austin, Austin, Texas 78712 USA; 4 Livestrong Cancer Institutes, Dell Medical School, The University of Texas at Austin, Austin, Texas USA; 5 Diagnostic Medicine, Dell Medical School, The University of Texas at Austin, Austin, Texas USA; 6 Oncology, Dell Medical School, The University of Texas at Austin, Austin, Texas USA

## Abstract

The development of resistance to chemotherapy is a major cause of treatment failure in breast cancer. While mathematical models describing the dynamics of resistant cancer cell subpopulations have been proposed, experimental validation has been difficult due to the complex nature of resistance that limits the ability of a single phenotypic marker to sufficiently identify the drug resistant subpopulations. We address this problem with a coupled experimental/modeling approach to reveal the composition of drug resistant subpopulations changing in time following drug exposure. We calibrate time-resolved drug sensitivity assays to three mathematical models to interrogate the models’ ability to capture drug response dynamics. The Akaike information criterion was employed to evaluate the three models, and it identified a multi-state model incorporating the role of population heterogeneity and cellular plasticity as the optimal model. To validate the model’s ability to identify subpopulation composition, we mixed different proportions of wild-type MCF-7 and MCF-7/ADR resistant cells and evaluated the corresponding model output. Our blinded two-state model was able to estimate the proportions of cell types with an R-squared value of 0.857. To the best of our knowledge, this is the first work to combine experimental time-resolved drug sensitivity data with a mathematical model of resistance development.

## Introduction

We aim to investigate how the therapeutic sensitivity of a breast cancer cell population changes over time following exposure to a pulse of chemotherapy. We hypothesize that intratumoral heterogeneity and cellular plasticity play a direct role in the progression of resistance. This hypothesis is based on previous work demonstrating that exposure to chemotherapy induces gene expression changes, metabolic state transitions, and increased drug resistance in subsets of cancer cells^1–10^. We test this hypothesis of the direct role of the changing composition of subpopulations of differing drug resistance in the observed resistance response using mathematical modeling to estimate the relative frequencies of cells in different drug sensitivity states over time.

Approximately 30 percent of women diagnosed with early-stage breast cancer develop resistance and ultimately progress to metastatic breast cancer^11^. Doxorubicin is a standard-of-care cytotoxic agent indicated for the treatment of breast cancer; however, the average time to develop resistance to doxorubicin is only 6 to 10 months^11^. Thus, it is critical to develop a mathematical-experimental approach to describe and predict the conditions and dynamics associated with the onset of resistance *in vitro*, ultimately to improve the efficacy of clinical treatment regimens. We and others have demonstrated evidence of cellular plasticity and adaptability in response to treatment with chemotherapy^1–6^. For example, it has recently been revealed that melanoma cells exhibit heterogeneity in their metabolic state, with cells utilizing different amounts of oxidative phosphorylation and aerobic glycolysis^9^. In this study of the role of metabolic usage in drug response, functional heterogeneity played a direct role in drug resistance as treating with a drug that inhibited aerobic glycolysis led to an increase in sensitivity to treatment^9^. The ability of individual cells to transition from a drug-sensitive to drug-resistant state has been observed in HL60 leukemia cells following chemotherapy exposure. Pisco *et al*. demonstrated that a subpopulation of cells increases expression of the ABC-transporter protein MDR1 in response to a chemotherapeutic pulse, leading to increased drug efflux and increased chemoresistance in those cells^1^. These experimental results focus on specific drug resistance phenotypes that emerge in cell subpopulations following treatment. However, because of the vast complexity of resistance mechanisms, it is difficult to identify a single molecular marker of drug resistance that encompasses all drug resistant cells^12,13^.

Mathematical descriptions of the dynamics of drug resistance may play a critical role in the development of strategies to combat drug resistance^12,14–18^. Theoretical models have been proposed that incorporate heterogeneous subpopulations in predicting and optimizing treatment response^19–28^ however, these models have not been fully validated with experimental cell population data *in vitro* or *in vivo*. While approaches that incorporate the heterogeneity of resistant and sensitive subpopulations are promising, they remain largely theoretical in nature^16^. Strategies such as optimal control theory^21,22^ (treatment aimed at maintaining the optimal composition of cell subpopulations), adaptive therapy^29^, and alternate metronomic dosing schemes^21,30^ have rarely been implemented in patient care because of lack of experimental validation. Validation of the presence of the predicted subpopulations proposed in these models is essential for progressing from theoretical predictions to implementation.

Although resistance to chemotherapy is a major cause of failure in breast cancer treatment, we do not currently have a mathematical model describing the development of resistance in the context of a dynamic heterogeneous cancer cell population. Conversely, experimental evidence concerning the variety of biological mechanisms of drug resistance is largely derived from static biological observations^31,32^. Many studies have relied on chemoresistant cell lines established by long-term exposure of cells to escalating doses of chemotherapeutic agent. In some cases, the chemotherapeutic is a required component of the cell culture media, to maintain resistant cell lines with a median lethal dose (LD50) up to 14 times higher than the original cell line^32^. Resistance observed in these cell lines may not be physiologically relevant to the clinical onset of chemoresistance, in which transient drug resistance may be induced in response to periodic treatment.

In this contribution, we calibrated experimental drug sensitivity data to multiple dynamic population models to test the hypothesis that there is a time-dependent population response to a chemotherapy treatment, and that this response is best described by models that incorporate heterogeneity and cellular plasticity. We combine the functional relevance of experimentally observed drug resistance data with various mathematical models to reveal the dynamic proportions of cells in subpopulations defined by their degree of drug resistance. To validate that our modeling approach was able to identify the composition of a cell population, we applied the model to known mixtures of reference cell populations with different resistance. To the best of our knowledge, this is the first effort to temporally resolve the proportion of drug sensitive and resistant cells in an experimental population in response to transient drug exposure.

## Materials and Methods

### Data acquisition

#### Cell culture

MCF-7 human breast cancer cells were obtained from ATCC and maintained in MEM (Minimum Essential Media, Thermo Fischer) supplemented with 10% fetal bovine serum (Gibco) and 1% Penicillin-Streptomycin (Gibco). MCF-7/ADR human breast cancer cells were obtained from Robert Clarke^33^ and maintained in MEM (Gibco) supplemented with 10% fetal bovine serum (Gibco), 1% Penicillin-Streptomycin (Gibco), and 500 nM doxorubicin (Sigma-Aldrich). A subline of the MCF-7 breast cancer cell line was engineered to constitutively express EGFP (enhanced green fluorescent protein) with a nuclear localization signal (EGFP-NLS). Genomic integration of the EGFP expression cassette was accomplished utilizing the Sleeping Beauty transposon system^34^. The EGFP-NLS sequence was ordered as a gBlock from IDT and cloned into the optimized sleeping beauty transfer vector pSBbi-Neo. pSBbi-Neo was a gift from Eric Kowarz (Addgene plasmid #60525)^34^. To mediate genomic integration, this two-plasmid system consisting of the transfer vector containing the EGFP-NLS sequence and the pCMV(CAT)T7-SB100 plasmid containing the Sleeping Beauty transposase was co-transfected into the MCF-7 population utilizing Lipofectamine 2000. mCMV(CAT)T7-SB100 was a gift from Zsuzsanna Izsvak (Addgene plasmid # 34879)^35^. GFP^+^ cells were collected by fluorescence activated cell sorting. This MCF-7-EGFPNLS1 cell line is maintained in MEM (Gibco) supplemented with 10% fetal bovine serum and 200 µg/mL G418 (Caisson Labs).

#### Time resolved resistance measurement

**Figure 1:**
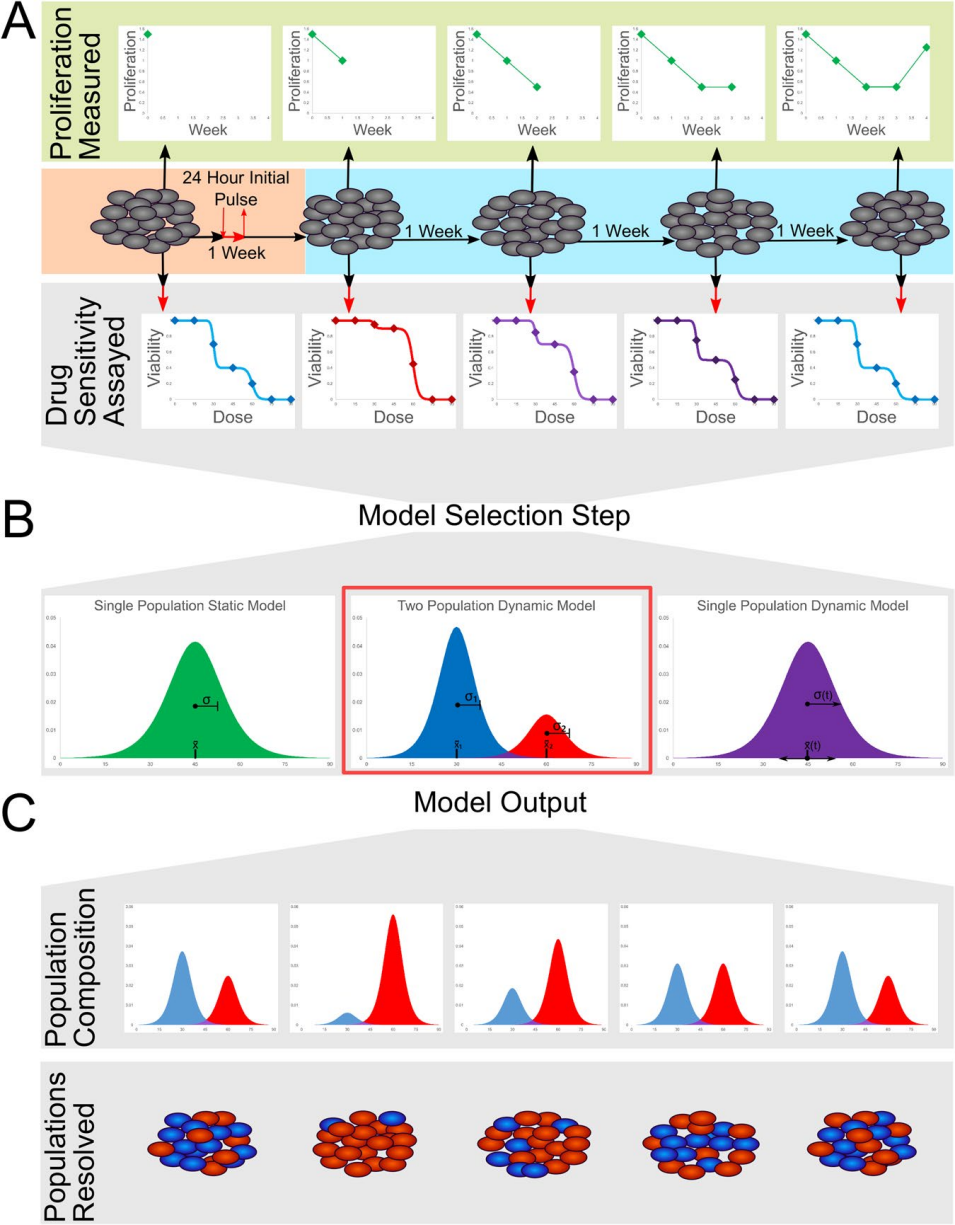
Experimental and modeling workflow: (**a**) MCF-7 breast cancer cells are treated with an initial pulse of doxorubicin (500 nM) for 24 hours. After treatment, the instantaneous growth rate is measured at each week. However, the subpopulation composition of drug sensitive and resistant cells is not easily identifiable from any single biomarker, as is indicated by the gray cells. To quantify the changes in drug resistance as the population responds to treatment, a subset of cells are extracted each week and a drug sensitivity assay is performed. (**b**) Using the combined data set containing a drug sensitivity assay at each time point, multiple mathematical models are tested to determine the optimal method for capturing the dynamic response of the cell population. Model selection statistics indicate that a multi-population model of at least two subpopulations is the optimal model. (**c**) The dynamic two population model estimates the presence of two subpopulations with distinct LD50s and variances corresponding to a sensitive and resistant subpopulation. The model mandates that these states remain constant throughout drug response, with the changes in drug sensitivity of the whole population resulting from changes in the proportions of the areas under the curve of the sensitive versus the resistant population. The model reveals the composition of resistant and sensitive subpopulations at each time point, as is indicated schematically by the ability to identify the proportions of red and blue cells in the population at each week.

MCF-7 cells were plated at 6600 cells/cm^3^ and cultured for two days in growth media. The media was then exchanged for growth media containing 500 nM doxorubicin. After 24 hours, the doxorubicin media was removed and replaced with growth media to end the drug pulse. Cells were passaged and counted weekly and drug sensitivity assays were performed weekly, as described below. Cell number counts at each week were used to determine the average per capita growth rate per day of the recovering cell population (Fig. 1A). The pulsed dosing of doxorubicin, followed by weekly drug sensitivity assays for 8 weeks, was repeated for a total of five independent MCF-7 cell populations in order to obtain multiple replicates at each time point assayed.

#### Weekly drug sensitivity assay

Each week, a subset (300,000) of the cells that were exposed to doxorubicin at the start of the experiment were plated into a 12-well plate in growth media. After two days of culture, media was exchanged for growth media containing doxorubicin at a range of concentrations (0, 4, 14, 24, 36, 48, 60, 72, 84, 96, 120, and 144 µM). Twenty-four hours after this dosing, the cells (including supernatant media) were collected via trypsinization, pelleted, and resuspended in 20 µL of media. Live and dead cells were identified with acridine orange and propidium iodide (ViaStain AOPI Staining Solution, Nexcelom Bioscience) and quantified with a Nexcelom Cellometer VBA. The ratios of live to dead cells were used to determine the viability at each concentration of doxorubicin (Fig. 1A).

#### Cell mixtures for model validation

MCF-7-EGFP-NLS1(wild-type) and MCF-7/ADR (resistant) cells were counted, mixed at desired ratios (1:0, 3:1, 1:1, 1:3, and 0:1), and plated in 12-well plates as described above (*Weekly drug sensitivity assay*). For each defined mixture, a sample of the untreated sample was counted in the Nexcelom Cellometer VBA to determine the measured percent of resistant cells, using the EGFP fluorescence of the MCF-7-EGFP-NLS1 cell line as a marker for the number of MCF-7-EGFP-NLS1 cells which are wild-type with respect to drug sensitivity. The measured percent of cells of each type was calculated by normalizing based on measurement of fluorescence in pure wild type (MCF-7-EGFP-NLS1) and MCF-7/ADR samples.

### Data Analysis

#### Calibration of experimental data to multiple structural models of dose-response

The combined data set containing the drug sensitivity assays from each week were fit to three different models (Table 1). The combined data set consists of three variables, the time (in weeks) post-initial doxorubicin exposure, the concentration of doxorubicin applied at that time, and the corresponding cell viability. To perform an estimation of the parameters for all three models, a nonlinear, least-squares approach was implemented in MATLAB (Mathworks). Sigmoidal viability curves are often used to describe chemotherapy dose-response curves^36^ because they correspond to a population of cells that die at different doses that can be described with a unimodal distribution of cells versus dose as shown in in Fig. 1B. The parameters of the sigmoidal dose response function are physically identifiable as the dose at which half the cells die (LD50) is the center parameter (*c*_*ss*_) and the standard deviation of the unimodal distribution is inversely related to the slope of the sigmoidal dose-response curve (*m*_*ss*_). The single static population model is the simplest of the models and ignores the temporal dependency of response. Here the drug response of the combined data set is described by a single homogenous cell population with a single (static) LD50 and slope. The single static population model equation is:

1 $${V}_{\sin gstat}(d)=\frac{{V}_{\max }}{1+\exp ({m}_{ss}(d-{c}_{ss}))}$$

where *V* is the proportion of cells viable at the dose, *d*, of doxorubicin in µM applied, *c*_*ss*_ is the LD50 of the population, *m*_*ss*_ is the slope at which the cells die due to increases in concentration, and *V*_*max*_ is the maximum viability of the cell population (as measured by the assay in absence of drug). The *V*_*max*_ parameter is included to normalize for naturally occurring cell death independent of the effects of doxorubicin. The single static model represents the null hypothesis that the initial pulsed dose has no time-dependency in its effect on the cancer cell population.

**Table 1:** Mathematical models to describe dynamic drug sensitivity data: We present the equations used for each of the three different structural models that were fit to the time-resolved drug sensitivity assays.

Description	Model equation	Variables and parameters
Single static model	${V}_{\sin gstat}(d)=\frac{{V}_{\max }}{1+\exp ({m}_{ss}(d-{c}_{ss}))}$	*V* = fraction of cells viable in population *V*_*max*_ = maximum viability of cell population (baseline viability at dose = 0 µM) *d* = dose of doxorubicin (µM) *m*_*ss*_ = slope of loss of viability as dose increases, *m*_*ss*_ = $\frac{1}{{\rm{\sigma }}}$ *c*_*ss*_ = LD50 to describe all data assuming no change in time *d* = dose of doxorubicin (µM)
Single dynamic model	${V}_{sindyn}(d,t)=\frac{{V}_{\max }}{1+\exp ({m}_{sd}(t)(d-{c}_{sd}(t)))}$	*m*_*sd*_(*t*) = slope of loss of viability as dose increases *m*_*sd*_(*t*) = $\frac{1}{{\rm{\sigma }}(t)}$ for each population *c*_*sd*_ (*t*) = LD50 to describe data at each time point
Two population dynamic model	${V}_{twopop}(d,t)={V}_{\max }(\frac{{f}_{sens}(t)}{1+\exp ({m}_{sens}(d-{c}_{sens})}+\frac{1-{f}_{sens}(t)}{1+\exp ({m}_{res}(d-{c}_{res})})$	*m*_*sens*_(*t*) = slope of loss of viability as dose increases, *m*_*sens*_(*t*) *=* $\frac{1}{{\rm{\sigma }}(sens)}$*, m*_*res*_(*t*) = slope of loss of viability as dose increases, *m*_*res*_(*t*) = $\frac{1}{{\rm{\sigma }}(res)}$*c*_*sens*_ = LD50 to describe sensitive population *c*_*res*_ = LD50 to describe resistant population

The column labeled, “Model equation” provides the functional form of the equation, with *t* representing a parameter that was fit to the data set at each time point measured. The column labeled, “Variables and parameters” describes the variables used in terms of their physical meaning and their relation to the time-resolved drug sensitivity assays.

The single dynamic population model incorporates a temporal dependency when fitting the combined data set. For each time point that drug sensitivity was assessed, the data is fit to an individual dose-response curve to generate LD50 and slope parameters. The model describes the drug response as a single homogenous population whose drug tolerance can change in time. The single dynamic population model equation is:

2 $${V}_{sindyn}(d,t)=\frac{{V}_{\max }}{1+\exp ({m}_{sd}(t)(d-{c}_{sd}(t)))}$$

where the *c*_*sd*_ and *m*_*sd*_ (LD50 and slope, respectively) parameters pertaining to each week, leading to a 16-parameter model (slope and LD50 at each of the 8 weeks). This model is akin to individually fitting a dose response curve to each week that the drug sensitivity assays were performed.

Finally, the two-population dynamic model describes a cell population with two cell states that differ in drug sensitivity. The dynamics of the drug response are captured by the relative frequency of cells in each state at each time point. The two-state dynamic population model equation is:

3 $${V}_{twopop}(d,t)={V}_{\max }(\frac{{f}_{sens}(t)}{1+\exp ({m}_{sens}(d-{c}_{sens}))}+\frac{1-{f}_{sens}(t)}{1+\exp ({m}_{res}(d-{c}_{res}))})$$

where each cell state is modeled as a subpopulation of cells whose LD50 is centered about a mean and slope (*c*_*sens*_, *m*_*sens*_ and *c*_*res*_, *m*_*res*_, respectively) which remain constant over time and whose *f*_*sens*_ and *f*_*res*_ (shown here as 1 − *f*_*sens*_) parameters can vary to best capture the drug sensitivity assay at each week. To fit the two-state model to data from multiple time points, the parameters of the sensitive and resistant slope and LD50 were forced to be constant at all time points, and the sensitive and resistant fraction parameters were allowed to float at each week, leading to a 12-parameter model (4 fixed parameters, 8 time-dependent fraction parameters for each of the 8 weeks). Equation 3 describes two cell states with distinct LD50 values whose relative frequencies are able to change in time after initial chemotherapy exposure, but whose LD50s remain constant. The overall cell population viability (measured) is modeled as a direct sum of the viability response in each subpopulation.

#### Statistical analysis and model selection

For all three models, the confidence intervals on the parameter estimates were constructed using the bootstrapping method of replacement^37^, with 500 bootstrapped simulated data sets. For each model, the mean-squared error and the Akaike Information Criterion^38^ were calculated for stand-alone model statistics. The Akaike Information Criterion estimator (i.e., the AIC value) is used for direct model comparison. The AIC value evaluates a model based on goodness of fit and penalizes for the complexity of the model using the number of parameters, with a lower AIC value indicating a better model. These evaluation criteria are used to determine the most appropriate model to describe the dynamic dose response data (Fig. 1b, Table 2).

**Table 2:** Model fit and model selection statistics indicate that the two population model is the optimal model: The two population dynamic model has the lowest AIC value and the lowest mean-squared error, indicating that the this model is superior to the single dynamic population model and the single static population model.

	Single static model	Single dynamic model	Two population model
Number of parameters	2	16 2 per time point	12 4 + 1 per time point
AIC value	−2004.4	−2015.6	−2126.4
Mean-squared error	0.057	0.040	0.024

The number of parameters for the single dynamic model and the two population model vary by the number of time-resolved drug sensitivity assays examined. In this case, for the single dynamic model there are two model parameters of LD50 and slope at each time point examined. In the two population model, there are four model parameters of LD50 and slope for the sensitive and resistant populations respectively, which remain constant, and one additional parameter per time point is used to describe the proportion of cells in each subpopulation at the time the cells were assayed.

#### Model validation

To validate the modeling approach, we tested each models’ ability to identify known mixtures of wild-type MCF-7 cells with MCF-7/ADR resistant cells. The same two population model and fitting algorithm described above (in the section labeled, *Calibration of experimental data to multiple structural models of dose-response***)** were used to fit the combined data set containing a mixture identifier, concentration of doxorubicin, and corresponding cell viability. Therefore, in this validation step, instead of grouping the data by week post drug treatment, we grouped by mixture composition. We allowed each group of mixture replicates to be fit to their own fractional parameter and maintained that the LD50 and centers of the two populations remain constant as described previously. We evaluated the model output of relative frequencies against our measured relative frequencies of wild-type MCF-7 and MCF-7/ADR cells (see *Cell mixtures for model validation*). The bootstrapping method of replacement, again with 500 simulated data sets, was used to construct the confidence intervals for each parameter estimate.

## Results

### Cancer cell population exhibits time-dependent response to pulse treatment

**Figure 2:**
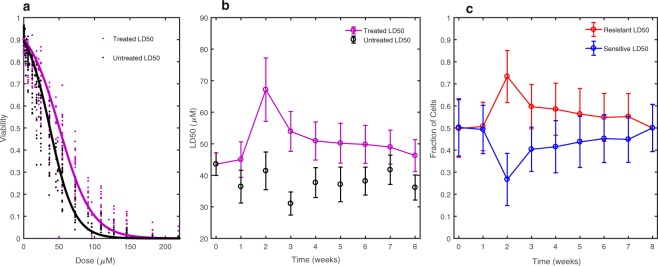
Time-resolved drug sensitivity assays fit to multiple models: (**a**) The static single population model (solid lines in panel a) demonstrates that the average resistance of the 8 weeks of compounded drug sensitivity data significantly increases from an LD50 = 37.0 +/− 3.5 µM for the untreated to an LD50 = 50.4 +/− 2.4 µM following exposure to pulse-treatment of doxorubicin. (**b**) The LD50 estimates resulting from analysis with the dynamic single population model indicate that the increase in resistance is time-dependent, peaking at 2 weeks after treatment with an LD50 = 67.2 +/− 10.0 µM, followed by a slow return towards baseline resistance levels, reaching an LD50 = 46.4 +/− 5.1 µM at 8 weeks. (**c**) The two population dynamic model displays the model-estimated proportions of a population with LD50 = 79.7 +/− 6.5 µM (resistant) and a population with LD50 = 22.4 +/− 2.0 µM (sensitive). The relative proportions of these two populations change over time, yielding the observed LD50 for the overall population. In panels b and c, parameter fits at each time point are connected by a line for visual aid and the error bars represent the 95% confidence intervals on the parameter values using the bootstrapping method of replacement with n = 500.

To determine whether the resistance of the MCF-7 population changes in time after the pulse treatment of doxorubicin, we fit the combined data set to both the static and dynamic single population models, as shown in Fig. 2a,b. In Fig. 2a, the experimentally measured viability is shown alongside the single static population model curve, for both the untreated controls (black) and the pulse-treated cell populations (purple). The LD50 to describe the resistance of these populations is 37.0 +/− 3.5 µM and 50.4 +/− 2.4 µM for the untreated and treated populations, respectively. Both slope and center parameters for the single static population model can be found in Table S1 of the Supplementary Materials. In Fig. 2b, we show the LD50 estimates, with the 95% confidence intervals, from the single dynamic model over all 8 weeks. The lower AIC value of the single dynamic model (−2015.6) compared to the single static model (−2004.4) indicates the time-resolved dose-response data are better described by the single dynamic model that allows the slope and LD50 value of the population to change at each time point (Table 2). The pattern in estimates of the LD50 values in the single dynamic model (Fig. 2b) corroborate this statistical analysis, showing a significant peak in the drug resistance at intermediate time points, with an LD50 at week 2 at 67.2 +/− 10.0 µM, followed by a slow return towards baseline, reaching 46.4 +/− 5.1 µM at 8 weeks. The LD50 and slope parameter values for the single static population model for all time points can be found in Table S2 of the Supplementary Materials.

### Incorporating heterogeneity via drug sensitivity states improves description of response

**Figure 3:**
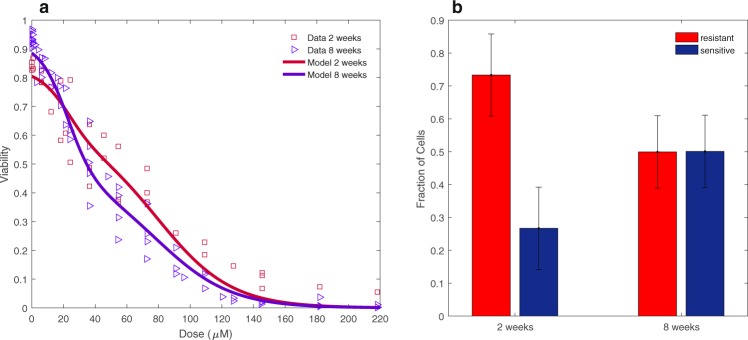
Example fit of drug sensitivity to the two-population model: (**a**) Best fit of the two- population model to the dose response data at two and eight weeks post-treatment demonstrates the ability of the model to capture the differences in subpopulation levels at the different time points. (**b**) Two population model output of parameter values of resistant and sensitive fractions at 2 and 8 weeks. The error bars represent the 95% confidence intervals on the parameter values using the bootstrapping method of replacement with n = 500.

To determine whether the dynamic drug response could be explained by a model of two subpopulations, we fit the data to the two population dynamic model to determine the degree of drug sensitivity of the two subpopulations and the resulting sensitive and resistant fraction parameter estimates at each week, with their 95% confidence intervals (Fig. 2c). The two-state dynamic model estimates the presence of a resistant subpopulation with an LD50 of 79.7 µM +/− 6.5 µM and a sensitive subpopulation with an LD50 of 22.4 +/− 2.0 µM (Fig. 2c). The parameter values for the sensitive and resistant slope and center, and the fractional parameters, can be found in Table S3 of the Supplementary Materials. We again use model selection to demonstrate that the two population model is an improvement over the single dynamic population model for describing the dynamic drug response of the cell population. Support for selection of this model is indicated by the lower AIC value (−2126.4) of the two population dynamic model compared with the single dynamic model (−2015.6) (Table 2). The results of the model selection analysis for describing dose response curves with multiple conditions (in this case time points) reveals that the differences in dose-response can not be modeled well by allowing each condition to be fit to a single sigmoidal curve allowed to shift, but rather is improved by modeling a sum of sigmoids corresponding to a multi-modal distribution of cells versus lethal dose whose proportions of cells in each state can change for each condition. To illustrate the improvement in fit to the data, Fig. 3a displays the two-state model curve overlaid on the dose-response data at 2 and 8 weeks, demonstrating the ability of the “shoulders” in the curve to fit the initially steep loss of cell viability at lower doxorubicin concentrations, as well as the persisting cell viability at higher doxorubicin concentrations. Figure 3b indicates the model output of relative frequencies of resistant and sensitive subpopulations for the model fit and data shown in Fig. 3a. The improvement in fit of the two-state model is additionally supported by the lower mean- squared error value for the two-state model than the single dynamic model (mean-squared errors of 0.024 and 0.040, respectively), despite it having less parameters than the single dynamic population model (Table 2).

### Subpopulation levels of resistant cells transiently increase

**Figure 4:**
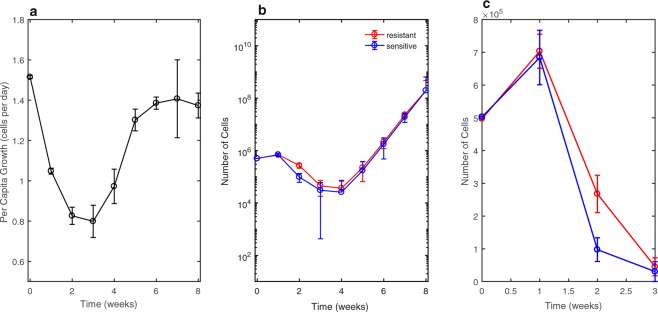
Data driven estimates of phenotypic dynamics: (**a**) Per capita growth rate (number of births per cell per day minus number of deaths per cell per day) of the entire cell population following pulse treatment of doxorubicin. Error bars represent the 95% confidence intervals from three replicates with per capita growth rate measured at each week. (**b**) Estimates of the number of resistant and sensitive cells over time are obtained by combining bulk population growth rate to estimate the total number of cells, with the estimates of subpopulation fractions from the two population model output. The estimates of the number of resistance and sensitive cells are purely empirical—cell numbers in each state can be obtained by a combination of differential growth rates, drug sensitivities, or cell state transitions and we do not attempt to identify the means by which the cell numbers are obtained. Error bars are the compounded 95% confidence interval of the per capita growth rate measurement and the parameter estimation of resistant fraction from the two population model output using the bootstrapping method of replacement with an n = 500. The number of cells is plotted in logarithmic scale. (**c**) A closer look in numeric scale of weeks 1–3 displaying the higher number of resistant cells over this time interval.

The key result for the treatment of doxorubicin on the MCF-7 cell line is that the proportion of cells in the resistant state is consistently higher than baseline from weeks 2 to 5 after the initial drug pulse, followed by return towards the initial resistant and sensitive subpopulation levels (Fig. 2c). The measured per capita growth rate per day (births per cell per day minus deaths per cell per day) (Fig. 4a) was used to estimate the total number of cells at each week. The number of total cells was combined with the fractional parameter estimates at each week (Fig. 2c) to obtain estimates of the number of resistant and sensitive cells at each week in the treated population (Fig. 4b,c). These estimates are purely empirical and make no assumptions about the mechanism at which the cells in each state reached the estimated number of cells at each time, allowing for the possibility of differential growth rates, drug sensitivities, and cell state transitions to determine the corresponding subpopulation levels. This differs from other published work which has assumed sensitive and resistant cells have different growth rates^29^ and distinct transition rates; here we do not attempt to define the mechanism by which the number of cells in each state was obtained.

### Model validation confirms ability to reveal subpopulation composition defined by drug sensitivity

**Figure 5:**
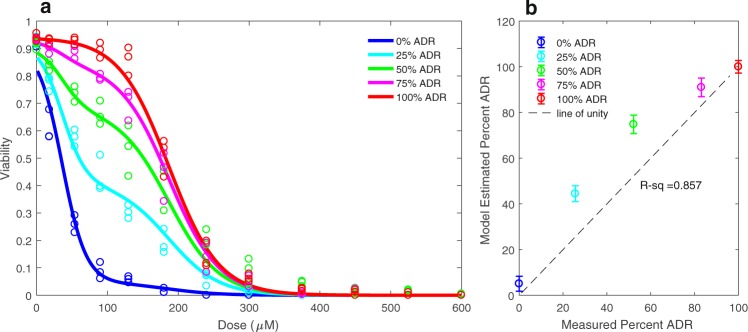
Model validation via identification of mixed populations: (**a**) The two-population model fit for each mixture of MCF-7/ADR resistant cell line and wild-type MCF-7 cell line overlaid on the experimentally measured drug sensitivity for the corresponding mixture. (**b**) Model-estimated percent of MCF-7/ADR cell line in mixture versus the measured MCF-7/ADR cell percentage using fluorescence cell counting. The coefficient of determination (R-squared) value of 0.857 indicates the efficacy of the fractional estimates from the two population model output.

Without a molecular marker of drug resistance, the estimated changes in drug resistant and drug sensitive subpopulations from our two-state model are difficult to validate; that is, we did not know for certain that our model estimated parameters of resistant and sensitive fractions reflect the true subpopulation compositions. We validated the modeling approach by generating experimental reference standards consisting of mixtures of the wild-type MCF-7 cell line with its corresponding doxorubicin resistant cell line, the MCF-7/ADR. We evaluated the ability of the model to estimate the subpopulation composition of each reference mixture. In Fig. 5a, the two population dynamic model fit for each mixture is overlaid on the dose-response data for the corresponding mixture. In Fig. 5b, the measured percent of wild-type and MCF-7/ADR cells are plotted as the line of unity against the parameter estimations of the percent of resistant cells from the model output (Table 3). The measured proportions of wild type and ADR cells versus the model output have a coefficient of determination (R-squared) of 0.857 (Fig. 5b).

**Table 3:** Model validation demonstrates identifiability of subpopulation compositions: Measured versus two population model estimated parameters indicate the model’s ability to reveal the known proportions of the MCF-7/ADR resistant cell line mixed with the wild-type MCF-7 cell line.

	Measured	Model
LD50_ADR_	187.5 +/− 3.8	186.7 +/− 4.6
slope_ADR_	0.034 +/− 0.0039	0.028 +/− 0.0028
LD50_WT_	37.1 +/−3.3	35.95 +/− 2.8
slope_WT_	0.055 +/− 0.0077	0.060 +/− 0.0076
frac_0%ADR_	0.07 +/− 0.01	0.05 +/− 0.03
frac_25%ADR_	0.30 +/− 0.04	0.44 +/− 0.04
frac_50%ADR_	0.54 +/− 0.05	0.075 +/− 0.04
frac_75%ADR_	0.83 +/− 0.05	0.91 +/− 0.04
frac_100%ADR_	1.0 +/− 0.02	1 +/− 0.0272

“Measured” LD50 values and slopes indicate the LD50 and slope of the isolated pure MCF-7/ADR cells and pure wild-type MCF-7 cells fit to the single static population model. Measured resistant fractions are measured precisely using the Nexcelom fluorescence imaging counter of the number of green fluorescent wild-type MCF-7 compared to the total number of brightfield cells counted.

## Discussion

The key results of this combined experimental and modeling approach demonstrate time-dependency in the response to a clinically relevant pulse-dose chemotherapy treatment, and a modeling system that reveals estimates of the composition of a cancer cell population with functional heterogeneity in its chemoresistance. We have identified that a transient increase in drug resistance is observed following drug exposure and have shown that this can be best described through a model that captures the changing compositions of distinct subpopulations defined by drug sensitivity. These subpopulations were not previously identified due to the complex nature of drug resistance mechanisms. The novelty in this modeling framework is that it is driven from drug sensitivity assays only, without imposing any assumed characteristics or isolated parameter measurements. A data-driven approach allows the model system to be applied to a variety of cell types and drug conditions; for example, in the model validation step, a mutant cell line with high resistance to doxorubicin is assessed. The key finding of this paper is to establish a foundation for describing observable resistance progression throughout time by revealing subpopulations that are not identifiable by molecular markers alone. Future work will need to systematically investigate the molecular and cellular mechanisms of these observed dynamics.

We acknowledge the many limitations of this study. The granularity of the modeling system was limited by technical constraints of the experiment dosing scheme. Bottlenecking of the population in the initial pulse-treatment limited the number of cells available at subsequent weeks for the corresponding drug sensitivity assay. While we were able to measure the response to 12 distinct doxorubicin concentrations each week, this was not sufficient to implement a multi-population model with more than two states, due to a lack of statistical power to significantly resolve differences in subpopulation compositions in time. We acknowledge that a model of only two subpopulations does not capture all likely relevant cell types but believe that this represents a useful simplification of resistance development. In the model validation phase, we were able to estimate the fractions of cells in each state with an R-squared value of 0.857 (Fig. 5b). We were also able to estimate the LD50s of the two populations in mixture using the model within the 95% confidence intervals of the isolated LD50s when fit to the static single population model alone (Table 3). It is possible that discrepancies in the fractional parameter estimates compared to the measured mixtures of the wild-type and resistant cell types may arise from biological interactions between the two that may increase the effective resistance of the cells when in contact with one another. A goal of future studies is to investigate the role of cell-cell interactions in drug resistance.

To ensure that the model parameters are uniquely identifiable and are not overfit to the experimental data sets, we tested the parameter identifiability of the model using simulated data with noise generated from the measured variability as a function of dose in the experimental data (Figure S1). We randomly generated 100 simulated data sets containing 5 different mixtures of resistant and sensitive cells to obtain a distribution for each of the parameter values, with the 95% confidence intervals around the true model parameter values shown in (Fig. S2), demonstrating that the variability in the estimated LD50 (Fig. S2a), slope (Fig. S2b), and fraction parameters (Fig. S2c) was reasonably small. We then addressed the question of the proximity at which the fractional parameters could be distinguished from one another by generating simulated data sets with experimental noise as described, this time containing either mixtures of mostly low levels of resistant cells (Fig. S3a) or of mostly high levels of resistant cells (Fig. S3b). In Fig. S3, the distribution of fractional parameter estimates is displayed as histograms, with each mixture labeled by color. We performed pairwise t-tests between each of the distributions of fractional parameter estimates to confirm that the distributions are statistically significantly different.

Experimental *in vitro* models of resistance to cytotoxic chemotherapy typically utilize resistant cell lines developed via continuous exposure to increased drug concentration^32,39^. In most cases, drug resistant phenotypes are characterized by an end-point analysis following the stabilization of a resistant cell population. Previous work in the field has indicated that the resistant phenotype of doxorubicin-resistant MCF-7 arises by a multi-factorial process because of observable differences in morphology, gene expression, and DNA content between MCF-7 and MCF-7 resistant cell lines^32^. The MCF-7/ADR resistant cell line used in our model validation step has an LD50 value of 187.5 +/− 3.8 µM (Table 3) and is thus more than 5 times more resistant than the wild-type MCF-7 with an LD50 value of 37.5 +/− 3.8 µM (Table 3). The MCF-7/ADR we used typically show a larger proportion of spindle-shaped cells that grow in a more dispersed manner than the wild-type cells^39^. Other groups have developed resistant MCF-7 cell lines that are 14-fold more resistant to doxorubicin than the original MCF-7 cell line^32^. They report that the MCF-7 resistant cells are on average larger, contain multiple nuclei, and upregulate genes involving metabolism, drug efflux, and down regulate genes involving DNA repair^32^. While these experimental observations provide us with key observables to identify as markers of resistance, they do not address the dynamic changes associated with resistance as it develops, nor are they all encompassing. To our knowledge, previous studies involving resistant cell lines have not reported time-resolved measurements of drug resistance following a clinically relevant pulsed dose chemotherapy treatment.

Mathematical modeling of heterogeneity in cancer cell populations has been investigated *via* multiple structural models^15–25^. In particular, many models have provided predictive capabilities of cell-line specific drug sensitivity^40^, as well as insightful metrics for capturing the growth inhibitory capacity of different drugs^23,41^. Explorations into different therapy strategies such as optimal control theory have utilized the concept of resistant and sensitive cells within a tumor or cancer cell population^17–25^. Many models of *in vitro* and *in vivo* cancer progression utilize compartmental ordinary differential equations and partial differential equations. In these models, oftentimes a number of key assumptions are made. For instance, in one model of a heterogeneous tumor, it is assumed that drug resistance is inversely related to proliferation rate^29^. Other models assume that all sensitive cells are susceptible to the chemotherapy, and do not account for the ability of initially sensitive cells to acquire drug resistance^16^. While these models can be extremely useful in capturing drug response and demonstrating the theoretical response to alternate treatment strategies under these sets of conditions, some of their predictions have yet to be fully validated experimentally due to technical limitations in identifying drug resistant subpopulation levels over time.

In this work, we reveal the dynamic changes in subpopulation composition in response to a pulse treatment of drug. In the future, time-resolved subpopulation relative frequencies can be used to develop a model that describes the relative stability of drug sensitivity states and how they change in response to chemotherapy exposure. Ultimately, the results of these experimentally guided models can be used to predict the effect, in terms of composition of resistant cells, of a specific dosing regimen on a cancer cell population over time. The goal of future studies is to use the proposed modeling framework to develop and experimentally validate optimal dosing regimens to be used to combat chemoresistance.

## Conclusion

We present this work as one demonstration of the role of heterogeneity in the development of drug resistance. Our analysis indicates that the response to pulsed chemotherapy is time-dependent and that the two-population model identifies subpopulation compositions that change over time. The approach we describe here uncovers chemoresistant subpopulations in breast cancer cell lines and is generalizable to any system in which subpopulations may play a role in a dynamic measurable outcome.

## Electronic supplementary material

Supplementary Information

## Data Availability

The complete datasets generated during and/or analyzed during the current study are available from the corresponding author on reasonable request.
