# Supplementary material for: A multi-state model of chemoresistance to characterize phenotypic dynamics in breast cancer
Source: Sci Rep. 2018 Aug 13;8:12058. doi: 10.1038/s41598-018-30467-w (PMC6089904; doi:10.1038/s41598-018-30467-w)
Supplement: Supplementary file 1 — Supplementary Information [file 41598_2018_30467_MOESM1_ESM.pdf]

Supplementary information

Multi-state model of chemoresistance to characterize phenotypic dynamics in breast cancer

Grant R. Howard[+], Kaitlyn E. Johnson[+], Areli Rodriguez Ayala, Thomas E. Yankeelov, Amy Brock*

[+] equal contribution
*Corresponding author: Amy Brock, amy.brock@utexas.edu

	Treated			Untreated		
	lower bound	value	upper bound	lower bound	value	upper bound
**LD50**	48.0	50.4	52.4	33.5	37.0	40.5
**slope**	0.038	0.044	0.049	0.045	0.0577	0.071

Table S1. Parameter values for the single static population model (LD50 values are in units of µM doxorubicin.)

	Treated			Untreated		
	lower bound	value	upper bound	lower bound	value	upper bound
**LD50** $_{t = 0wk}$	40.1	43.6	47.1	40.2	43.6	47.2
**LD50** $_{t = 1wk}$	39.6	45.0	50.4	31.2	36.4	41.6
**LD50** $_{t = 2wk}$	57.1	67.1	77.1	35.4	41.4	47.4
**LD50** $_{t = 3wk}$	47.9	53.9	60.0	27.4	31.0	34.7
**LD50** $_{t = 4wk}$	44.8	50.9	57.0	32.9	37.7	42.4
**LD50** $_{t = 5wk}$	43.8	50.2	56.5	31.6	37.1	42.6
**LD50** $_{t = 6wk}$	43.8	49.8	55.7	33.7	38.2	42.6
**LD50** $_{t = 7wk}$	43.6	48.9	54.3	37.1	41.8	46.4
**LD50** $_{t = 8wk}$	40.8	46.2	51.7	32.1	36.1	40.1
**slope** $_{t = 0wks}$	0.039	0.052	0.065	0.039	0.052	0.065
**slope** $_{t = 1wks}$	0.031	0.051	0.069	0.036	0.057	0.077
**slope** $_{t = 2wks}$	0.020	0.031	0.043	0.032	0.049	0.067
**slope** $_{t = 3wks}$	0.026	0.039	0.052	0.046	0.064	0.083
**slope** $_{t = 4wks}$	0.030	0.045	0.060	0.039	0.057	0.074
**slope** $_{t = 5wks}$	0.030	0.045	0.060	0.036	0.053	0.070
**slope** $_{t = 6wks}$	0.029	0.041	0.054	0.043	0.060	0.076
**slope** $_{t = 7wks}$	0.035	0.049	0.063	0.039	0.056	0.072
**slope** $_{t = 8wks}$	0.035	0.049	0.064	0.43	0.064	0.084

Table S2. Parameter values for the single dynamic model (LD50 values are in units of µM doxorubicin.)

	Treated			Untreated		
	lower bound	value	upper bound	lower bound	value	upper bound
LD50 $_{res}$	73.2	79.7	86.3	73.2	79.7	86.3
LD50 $_{sens}$	19.2	22.4	25.6	19.2	22.4	25.6
slope $_{res}$	0.037	0.043	0.050	0.037	0.043	0.050
slope $_{sens}$	0.073	0.115	0.158	0.03	0115	0.0158
frac$_{res\ t=0wk}$	0.379	0.497	0.626	0.379	0.497	0.626
frac$_{res\ t=1wk}$	0.406	0.507	0.608	0.280	0.381	0.483
frac$_{res\ t=2wks}$	0.597	0.733	0.869	0.356	0.464	0.572
frac$_{res\ t=3wks}$	0.500	0.597	0.693	0.174	0.270	0.367
frac$_{res\ t=4wks}$	0.473	0.585	0.697	0.286	0.392	0.500
frac$_{res\ t=5wks}$	0.460	0.563	0.667	0.279	0.381	0.483
frac$_{res\ t=6wks}$	0.448	0.548	0.648	0.310	0.406	0.502
frac$_{res\ t=7wks}$	0.449	0.551	0.653	0.350	0.464	0.578
frac$_{res\ t=8wk}$	0.395	0.499	0.603	0.256	0.361	0.465

Table S3. Parameter values for the two-population model (LD50 values are in units of µM doxorubicin.)

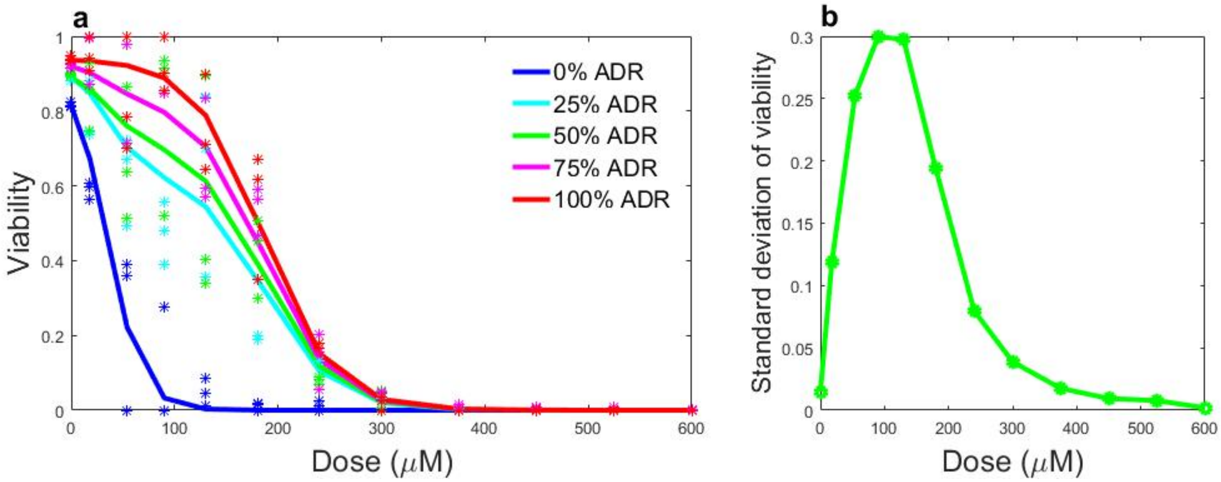

Figure S1. Two-population model and an example simulated data set with experimental noise. a. We inputted model parameters to simulate a resistant population with an LD50 of 185 µM and a sensitive population with an LD50 of 35 µM, and resistant fractions from 0-100 %, and simulated data with noise added according to experimentally distributed noise. **b.** This plot indicates the standard deviation in the measured cell viability of all mixtures of the naïve MCF-7 cells and the MCF-7/ADR resistant cells as a function of dose. The observed variability in

viability measurements shown here were used to simulate data sets with experimentally observed noise. This plot indicates that the highest variability in cell survival response occurs at intermediate doses.

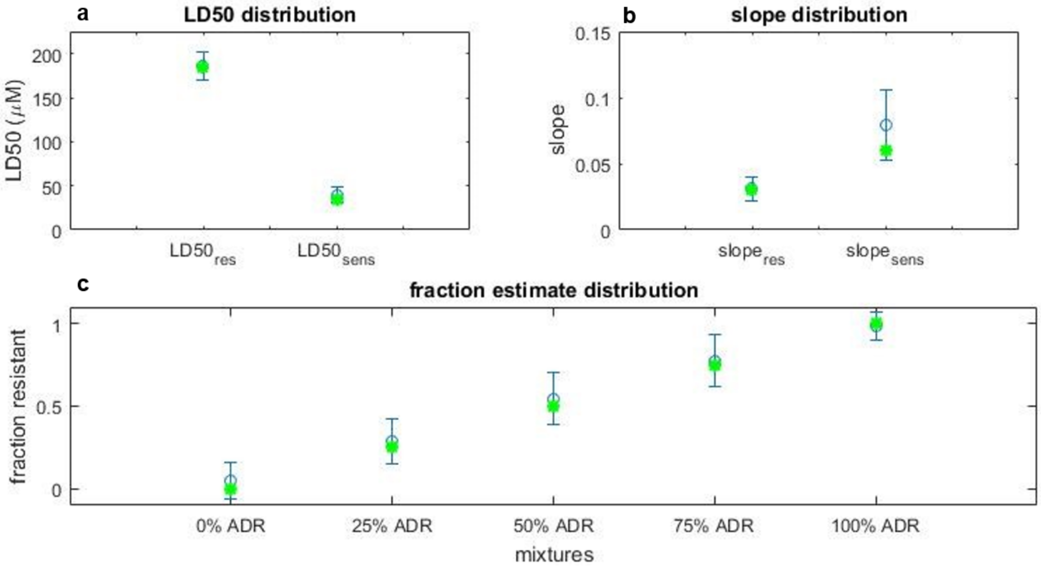

Figure S2. Parameter distributions for fitting of simulated data sets with experimental noise. a. We display the ninety-five percent confidence intervals around 100 fitted LD50 sensitive and resistant parameters for the simulated data sets in order to demonstrate the relative error in parameter identifiability of the LD50 values at the experimental noise observed. **b**. We display the ninety-five percent confidence intervals around 100 fitted sensitive and resistant slope parameters for the simulated data sets in order to demonstrate the relative error in parameter identifiability of the slope values at the experimental noise observed. We observe a higher relative error in the sensitive slope than in the resistant slope. **c**. We display the ninety-five percent confidence intervals around 100 fitted fraction estimate parameters for the simulated data sets in order to demonstrate the relative error in parameter identifiability of the fractions at the experimental noise observed. We observe the error to be fairly consistent across all of the mixtures.

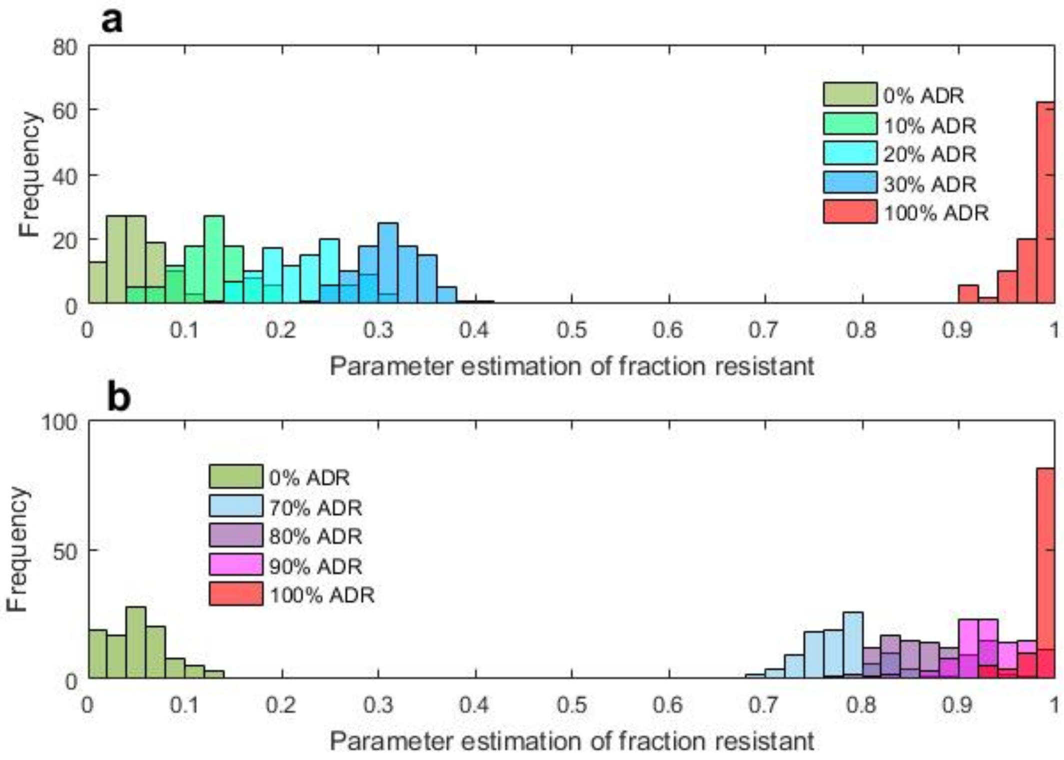

Figure S3: Parameter distributions around clustered input fraction parameters are identifiable within 10% with current experimental noise. a. Model parameters of clustered low resistant fractions were input at intervals of 10% from 0-30% resistant. One hundred simulated data sets with experimental noise were fit to obtain 100 model parameter estimates for each fractional parameter. We compared each pairwise set of low resistant fraction parameter distributions with a multiple comparison t-test in Matlab and found significant differences with a p-value of 9.92×10^{-8}. **b.** The same procedure was performed for high resistant fractions at intervals of 10% increase from 70-100% resistant. Again, the distributions of high resistant fraction parameter estimates were significantly different with a p-value of 9.92×10^{-8}.

\

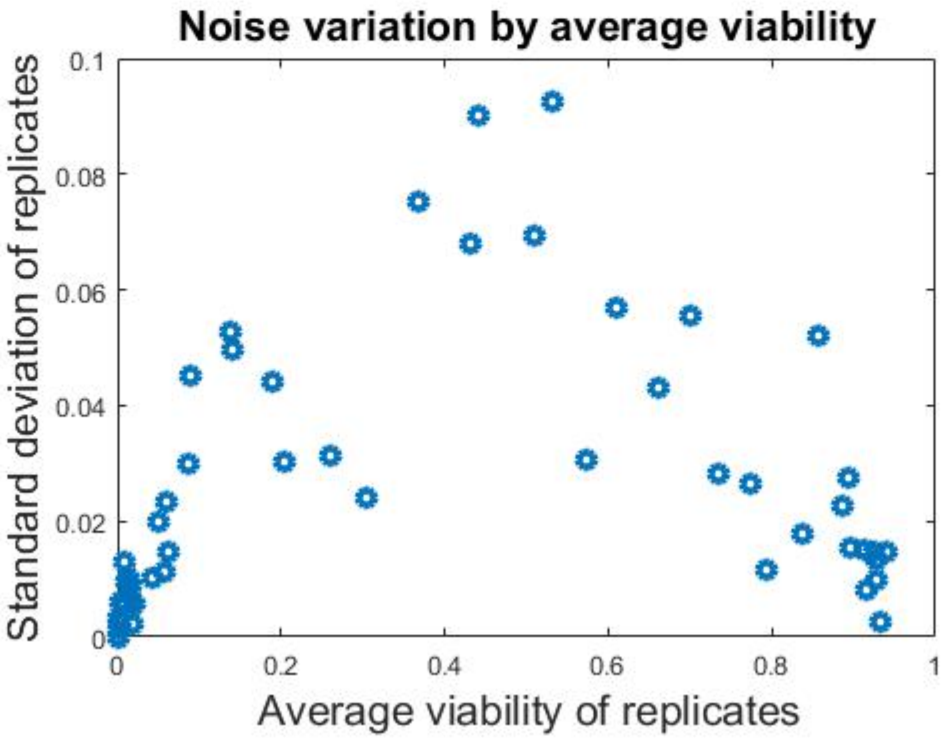

Figure S4: Data from the model validation experiment is used to estimate sources of error. The standard deviation among each set of replicate measurements is graphed against its average value, with each set of replicates represented as a single point.

Discussion of Intrinsic Stochastic Biological Variability and Sources of Error

While we often look at variation in measured data and mentally summarize it as "measurement error", the controls present in our model validation experiment allow us to decompose this variation into several sources of error. Error in the viability measurement performed using the Nexcelom Cellometer is approximately 0.5%, estimated using replicate measurements drawn from a single pool of cells. The variation in viability is larger, at an average of 2.2% across all samples, but is also non-uniformly distributed, as shown in **Figure S4**.

Part of this variation can be explained as the result of differences in the proportion of cells of each subline plated into a given well; this variation has a standard deviation of approximately 1.7%. In **Figure S4,** however, the standard deviation ranges as high as 9%, indicating that an additional source of variation is present, and appears to be dependent on the

population viability for that set of replicate measurements. We believe that this is best described as an actual variability in the response that replicate populations will display when given the same stimulus, due to downstream effects of stochastic variation in the early response. Among other things, we are aware that confluence has a large effect on cell survival – as a result, random fluctuations in early survival could snowball into these substantial differences over the duration of the drug perturbation. As each cell dies, it increases the probability that other nearby cells will die. (We speculate that this is mediated by the loss of pro-survival signals which the cells exchange.) Any fluctuation of survival in the early stages of the response is then propagated and amplified, magnifying the fluctuations into the pattern of variation seen here in supplemental **Figure S4**. This theory is consistent with the non-uniform distribution of variability; doses that are high or low enough to have more deterministic effects show minimal variation, more comparable to the error known to be present from variation in the initial seeding proportions and technical error in the assay while in doses where the probability of death is closer to 50%, a marginal change in the probability of survival is more likely to influence the outcome.
